# Chitosan-Graft-Polyethylenimine/DNA Nanoparticles as Novel Non-Viral Gene Delivery Vectors Targeting Osteoarthritis

**DOI:** 10.1371/journal.pone.0084703

**Published:** 2014-01-02

**Authors:** Huading Lu, Yuhu Dai, Lulu Lv, Huiqing Zhao

**Affiliations:** Department of Orthopedics, Third Affiliated Hospital of Sun Yat-sen University, Guangzhou, P. R. China; The Ohio State University, United States of America

## Abstract

The development of safe and efficient gene carriers is the key to the clinical success of gene therapy. The present study was designed to develop and evaluate the chitosan-graft-polyethylenimine (CP)/DNA nanoparticles as novel non-viral gene vectors for gene therapy of osteoarthritis. The CP/DNA nanoparticles were produced through a complex coacervation of the cationic polymers with pEGFP after grafting chitosan (CS) with a low molecular weight (Mw) PEI (Mw = 1.8 kDa). Particle size and zeta potential were related to the weight ratio of CP:DNA, where decreases in nanoparticle size and increases in surface charge were observed as CP content increased. The buffering capacity of CP was significantly greater than that of CS. The transfection efficiency of CP/DNA nanoparticles was similar with that of the Lipofectamine™ 2000, and significantly higher than that of CS/DNA and PEI (25 kDa)/DNA nanoparticles. The transfection efficiency of the CP/DNA nanoparticles was dependent on the weight ratio of CP:DNA (w/w). The average cell viability after the treatment with CP/DNA nanoparticles was over 90% in both chondrocytes and synoviocytes, which was much higher than that of PEI (25 kDa)/DNA nanoparticles. The CP copolymers efficiently carried the pDNA inside chondrocytes and synoviocytes, and the pDNA was detected entering into nucleus. These results suggest that CP/DNA nanoparticles with improved transfection efficiency and low cytotoxicity might be a safe and efficient non-viral vector for gene delivery to both chondrocytes and synoviocytes.

## Introduction

Osteoarthritis (OA) is one of the greatest challenges for clinical therapy due to avascularity and the lack innervation of cartilage. Current therapy shows little effects because of the rapid clearance of therapeutic agents by the synovium and the difficulty of infiltrating into the dense extracellular matrix (ECM) [1]. Gene therapy represents a promising technology for OA treatment by targeting specific disease-relevant mechanisms, and thus by treating the causes of OA rather than the symptoms [2]. The development of an efficient and safe gene transfer system is one of the most important factors for a successful gene therapy with practical use in the clinic. Although most gene therapy protocols in clinical trials actually employ viral vectors due to their high transfection efficiency, their fatal drawbacks, such as immunogenicity, potential infectivity, complicated production, and oncogenic effects, may prevent their further use in the clinic [3]–[6]. On the other hand, non-viral vectors have attracted much attention because of their potential advantages, such as ease of synthesis and modification, efficient cell/tissue targeting, low immune response, and unrestricted plasmid size, among others [4]–[9].

Among various non-viral vectors, chitosan (CS) is considered to be an excellent candidate, because of its biocompatibility, biodegradability, lower toxicity, and higher cationic potential properties [10]–[12]. CS/DNA complexes have been shown to be capable of transfecting chondrocytes under *in vitro* and *in vivo* conditions [2]. However, they also have low transfection efficiency, thus limiting its further application as a non-viral gene delivery vector [7], [9], [12]–[15]. There is an increasing interest in improving CS properties by various modifications. In our previous study [16], we designed hybrid hyaluronic acid (HA)/CS-plasmid nanoparticles as novel non-viral gene delivery vectors targeting OA. In this study, HA/CS-plasmid nanoparticles demonstrated significantly higher transfection efficiency towards chondrocytes compared to CS/DNA complexes. HA-containing nanoparticles gain facilitated access to target cells via receptor-mediated endocytosis pathway through HA-CD44 interaction, and this interaction also leads to a cellular signaling process, which could promote the success of the gene transfection [16]. However, the transfection efficiency of HA/CS-plasmid nanoparticles in synoviocytes has been demonstrated to be very poor (unpublished data), which may restrict their future clinical application. The unsatisfactory transfection efficiency of either CS or its derivatives is mainly a result from its poor endosomal escape due to lack of buffering amines [9], [17], and also from the very strong binding with DNA resulting in inefficient unpackaging of genetic material in the cytoplasm [9], [18].

As reported, the transfection efficiency CS vectors can be improved by combining CS with either cationic or anionic biopolymers, such as polyethylenimine (PEI) [8], [9], [19], prior to the addition of DNA. PEI is another promising cationic non-viral vector, thanks to its high buffering capacity. PEI can protect DNA from nuclease degradation and facilitate endosomal escape of PEI/DNA complexes [8], [9], [15], [20], [21]. However, the high toxicity *in vitro* and *in vivo* of this non-viral vector limits its application in gene therapy [5], [6], [8], [9], [21]. Fortunately, many pilot studies had proved that the combination of CS and PEI (CS/PEI blend or CS-graft-PEI -CP) can simultaneously enhance the transfection efficiency and decreasing the cytotoxicity [4], [8], [9], [13]–[15]. However, most of these studies were carried out in tumor cells such as HeLa, A549, and HepG2 cells [8], [9], [13]–[15], [22]. Consequently, it is unknown how the novel non-viral vector behaves in both chondrocytes and synoviocytes, which is important for us to determine its value in gene therapy for OA or other joint diseases. To address the limitations of HA/CS-plasmid nanoparticles and explore an effective non-viral gene vector focused on OA, we deliberately selected a low Mw PEI (1.8 kDa), which possesses an adequate buffering capacity with poor transfection efficiency. For its part, both PEI and CS are biodegradable [8].

Usually the gene therapy for OA select chondrocytes as target cells, which enables the function of gene expression products through autocrine and paracrine mechanisms [2]. However, since chondrocytes are wrapped around the dense cartilage matrix it is quite difficult for a non-viral gene vector to infiltrate into the dense ECM and thus to reach the deep cartilage. In addition to the destruction of articular cartilage and the formation of osteophytes, OA is accompanied with chronic inflammation of the synovium, which plays an important role in the occurrence and development of the disease [23], [24]. Synovium is widely distributed in the intra-articular joint cavity, and directly contacts with the articular cartilage in some place; its secreted synovial fluid provides nutrition and exchange material with articular cartilage. Gene therapy towards synoviocytes enables gene expression products to reach the cartilage surface through synovial fluid, thus affecting the metabolism of the cartilage and reversing the OA progression. Therefore, gene therapy targeting the synoviocytes may be more effective than targeting other cell types for either OA or other joint diseases [25], [26].

In the present study, a novel CS/DNA complex grafted with low Mw PEI was prepared as a new non-viral gene carrier for the OA-targeted intracellular delivery of therapeutic genes into chondrocytes and synoviocytes. We investigated the characteristics of CP/plasmid enhanced green fluorescent protein (pEGFP) nanoparticles and their cytotoxicity, the transfection efficiency in chondrocytes and synoviocytes, and their ability to carry nucleic acids into the nucleus of chondrocytes and synoviocytes. Our results will facilitate the assessment of CP/DNA nanoparticles feasibility as an efficient and safe non-viral vector to deliver therapeutic genes to chondrocytes and synoviocytes for the treatment of either OA or other joint diseases.

## Materials and Methods

### Materials

CS (Mw = 50 kDa and deacetylation degree = 90%), PEI (Mw = 1.8 kDa and 25 kDa), and 4', 6-diamidino-2-phenylindole (DAPI) were purchased in Sigma-Aldrich (St. Louis, MO, USA). Dulbecco’s modified Eagle’s medium (DMEM), D-Hanks, 0.25% trypsin/ethylenediaminetetraacetic acid (EDTA), penicillin, and streptomycin were obtained from Gibco-BRL (Gaithersberg, MD, USA). The fetal bovine serum (FBS) was obtained from HyClone (Logan, UT, USA). The Cell Counting Kit-8 (CCK-8) was purchased in Dojindo (Kumamoto, Japan). The 1,1′-carbonyldiimidazole (CDI) was obtained from Pierce (Rockford, IL, USA). The pReceiver-M29 vector carrying an EGFP, Lipofectamine™ 2000, and LysoTracker Green DND-26 were purchased in Invitrogen (Carlsbad, CA, USA). The Cy3-labeled plasmid DNA (pDNA) was made by RiboBio (Guangzhou, P. R. China). The plasmid was propagated in *Escherichia coli* cells, isolated, and then purified.

### Synthesis of Copolymer

Copolymer of CP was prepared according to the methods described by Gao *et al*. [8]. Briefly, 0.5 g CS was dissolved in 20 ml of a 0.5% acetic acid solution, which was then adjusted at pH 7.0, and the mixture was stirred overnight. CDI was then added at a molar ratio of CDI:CS amine = 2∶1, followed by stirring for 1 h. The PEI (1.8 kDa) was added dropwise into the solution under stirring at a molar ratio of PEI:amine = 2∶1. The polymerization was performed at room temperature (25°C) overnight. The resultant product was purified by dialysis in water for 48 h. Finally, the resultant CP powder was collected by lyophilization.

### Characterization of Copolymer

The composition of the prepared CP copolymer was analyzed by proton nuclear magnetic resonance spectroscopy (^1^H NMR). CS and CP were dissolved in the mixed solvent D_2_O/CD_3_COOD (V_D2O_: V_CD3COOD_ = 1∶1). ^1^H NMR spectra were recorded using a Bruker AV600 spectrometer (Advance TM 600, Bruker, Germany). The molecular weights and molecular weight distributions of CS and CP were measured by using a Gel permeation chromatography (GPC) system (Malvern, Viscotek GPC Max VE 2001, MA, UK) equipped with a Viscotek TDA 305 Triple detector array, a Malvern CLM 3021 A6000M column using 0.2 mol/l HAc/NaAc buffer as the eluent at a flow rate of 0.5 ml/min at 40°C. Polymer samples were dissolved in 0.2 mol/l of HAc/NaAc to form the 2.5 mg/ml solution and filtered through a 0.45-µm syringe filter, and 100 µl of polymer solution was injected into the GPC system. The molecular weights of the polymer samples were analyzed by using OmniSec 4.6.0 software.

### Preparation of CP/DNA Complex

All CP/DNA nanoparticles were freshly prepared before use. The principle of nanoparticle formation was mutual attraction between positive and negative charges. The CP powder was dissolved in distilled water and filtered through a 0.22 µm membrane. Then, CP/DNA nanoparticles were prepared at various CP:DNA weight ratios (0.5, 1, 2, 3, 4, and 5) by gently dropwise adding the copolymer solution to equal volumes of DNA solution (1 µg/µl). The mixture was vortexed gently for 5 min and left at room temperature for 30 min to form CP/DNA nanoparticles solution. The charge ratio (N/P) of CP/DNA complexes are defined as the molar relation of amine groups in the cationic molecule of CP complex to phosphate groups in the DNA. For calculation of N/P ratios, 330 Da was used as an average mass per charge for DNA [8].

### Scanning Electron Microscopy (SEM)

Nanoparticles in solution were dropped onto a silica surface, lyophilized using a JFD-310 (JEOL, Japan), then precoated with a thin layer of gold and palladium prior to analysis. Micrographs were obtained using a SEM (JSM-6360, Japan).

### Particle Size and Zeta Potential Measurements

A Mastersizer 2000 laser diffractometer (Malvern Instruments, Worcestershire, UK) was used to measure the particle size and the surface charge (represented by the surface zeta potential) of the test nanoparticles prepared and analyzed in distilled water at 25°C. The volume of samples was 1 ml containing a final DNA concentration of 50 µg/ml.

### Gel Retardation Assay

CP/DNA nanoparticles were evaluated by agarose gel electrophoresis (AGE). Nanoparticles were prepared at different weight ratios of CP:DNA of 1∶2, 1∶1, 2∶1, 3∶1, 4∶1, 5∶1, 6∶1, and 7∶1. The effect of CP on DNA condensations was investigated by electrophoresis on a 1% agarose gel containing ethidium bromide in Tris-borate EDTA buffer at pH 6.5. The samples were run on a gel at 100 V for 30 min. The resultant DNA migration pattern was revealed under a GDS-8000 (UVP, USA).

In order to determine whether the combination between CP and pDNA was affected by pH value, CP/DNA nanoparticles samples (e.g., CP:DNA weight ratios = 3) were evaluated under different pH conditions (5.5 to 8.0) by AGE as described above. Similarly, CS/DNA nanoparticles (e.g., CS:DNA weight ratios = 3) were evaluated and used as control under the same pH conditions.

### Analysis of Buffering Capacity

The buffering capacity of different complexes (CS, PEI -25 kDa-, and CP) was compared by acid titration experiments, with the NaCl solution (0.1 N) as negative control. Briefly, CS, PEI (25 kDa), and CP copolymers were separately dissolved in 0.1 M NaCl to obtain the final concentration of 0.1 mg/ml, and the pH of each solution was adjusted to pH10 with 0.1 N NaOH. Then, 0.1 M HCl (∼20 µl per drop) was added dropwise to sample solutions. Different pH values were measured using a pH meter. The slope of the line in the plot for pH against the amount of HCl consumed indicated the intrinsic buffering capacity of the system [27], [28].

### Animals and Cell Culture

All procedures involving animals in this study were reviewed and approved by the Institutional Animal Care and Use Committee at the Sun Yat-sen University (Guangzhou, P. R. China) (Approval ID: 2011-0905). Cartilage and synovial tissues were harvested under sterile conditions from knee joints of three-week-old New Zealand white rabbits (n = 6; Laboratory Animal Center of Sun Yat-Sen University, Guangzhou, P. R. China; Animal quality certificate numbers: SCXK 2008-0002).

Cartilage slices were treated at 37°C with 0.25% trypsin for 30 min, thoroughly washed with phosphate-buffered saline (PBS), and incubated with 0.2% (w/v) collagenase II (activity 277.0 units/mg; Gibco-BRL, Gaithersberg, MD, USA) at 37°C for 12 h. After digestion of tissues with trypsin and collagenase II, the cells were harvested every 2 h for a total of 4–6 times, and isolated chondrocytes were cultured in monolayer in DMEM supplemented with 10% FBS, 100 µg/ml of streptomycin and 100 U/ml of penicillin at 37°C and 5% CO_2_.

Synoviocytes were isolated and cultured according to the method previously described [29]. Briefly, synovial tissues were minced aseptically into 1–2 mm^2^, and then digested in DMEM containing 5% (v/v) FBS and 0.2% (w/v) collagenase II at 37°C and 5% CO_2_ for 2 h. The adherent cells were discarded, and non-adherent tissues were digested in serum-free DMEM containing 0.25% trypsin for 30 min. Then, they were transferred through sterile 108 µm^2^ nylon mesh into a sterile centrifuge tube, and centrifuged at 300×g for 10 min. Resulting cells were washed extensively with DMEM, and then cultured in DMEM supplemented with 10% (v/v) FBS, 100 U/ml penicillin, and 100 µg/ml streptomycin at 37°C and 5% CO_2_. At confluence, adherent cells were trypsinized, split in a 1∶3 ratio, and recultured in medium.

### Cytotoxicity Assay

*In vitro* cytotoxicity tests were performed by the CCK-8 assay [16]. Chondrocytes and synoviocytes were seeded at a density of 5×10^4^ cells per well in 100 µl of culture medium in 96-well plates, and incubated for 24 h to reach 80% confluence at treatment. Immediately after culture medium was removed, CP/DNA, CS/DNA, and PEI (25 kDa)/DNA nanoparticles (weight ratio = 3), at polymer concentrations ranging from 5 to 40 µg/ml in fresh culture medium without serum, were added to cells. In another group of cells, culture media were replaced by fresh serum-free media containing Lipofectamine™ 2000 (5 µg DNA/ml) as positive controls. A group of cells treated with only fresh culture medium was used as blank controls. Cells were incubated with various complexes for 4 h; various media were then removed and replaced with fresh culture media. Then, medium containing CCK-8 (10 µg per well) was added, and cells were maintained in an incubator at 37°C and 5% CO_2_ for 3 h. Optical density was measured using a microplate reader (Bio-RAD, model 680) at a wavelength of 570 nm, using a blank control consisting of CCK-8 solution. Three replicates were performed for each sample and the mean value was reported as final results.

### *In vitro* GFP Transfection Experiment

Chondrocytes and synoviocytes were seeded into 24-well plates at a density of 1×10^5^ cells per well in 500 µl of culture medium, and incubated for 24 h. For the transfection assay, the medium was discarded and cells were washed once with PBS. Nanoparticles containing pEGFP (CP/DNA nanoparticles with CP:DNA weight ratio = 3 were used as example) were added to cells in FBS-free DMEM, which were then incubated for 4 h. The nanoparticles solution was removed and replaced with fresh culture medium supplemented with serum and antibiotics. After being cultured for 48 h, EGFP-positive cells were detected using a fluorescence microscope (Nikon-TE2000U, Tokyo, Japan).

Cells were collected and resuspended in PBS (pH 7.4), and transfection results were measured using a fluorescence-activated cell sorting (FACS) device (Calibur, BD, USA) through the first fluorescence channel. Cells which were exposed to naked pDNA, CS/DNA nanoparticles, or PEI (25 kDa)/DNA nanoparticles (weight ratio = 3) were analyzed using FACS as controls (at a pEGFP concentration of 4 µg/ml and incubated for 4 h in FBS-free DMEM, respectively). Lipofectamine™ 2000 was used as a positive control for transfection study, and was added to DMEM without serum or antibiotics, following manufacturer procedures. Then, Lipofectamine™ 2000 was incubated with 0.8 µg pEGFP per well for 4 h. Posteriorly, the medium was discarded and replaced with a complete medium containing serum and antibiotics for 48 h, according to instructions. All transfection experiments were performed in triplicate.

To evaluate the transfection efficiency of CP/DNA with different CP:DNA weight ratios, nanoparticles containing pEGFP (CP:DNA weight ratio range from 1∶2, 1∶1, 2∶1, 4∶1, 8∶1 to 10∶1) were added to cells (with a concentration of 2 µg DNA per well) in FBS-free DMEM, and incubated for 4 h at 37°C and 5% CO_2_, as described above. The transfection efficiency was determined by fluorescent microscopy and FACS, as aforementioned.

### Intracellular Trafficking

Prior to treatment, either chondrocytes or synoviocytes were seeded at a density of 1×10^5^ cells/dish in a culture medium containing 10% FBS for 24 h. When cells were cultured at 70% confluence, the medium was removed and cells were washed twice with PBS. Before adding 300 µl treatment solution containing LysoTracker Green DND-26 (50 µM) (Invitrogen, Carlsbad, CA, USA) in FBS-free DMEM for the mixture the medium was incubated for 20 min to label lysosomes. After removal of the medium, the cells were washed with PBS twice. Subsequently, CP/DNA nanoparticles containing 2 µg Cy3-labeled pDNA (RiboBio, P. R. China) were diluted in 200 µl of serum-free DMEM medium, and were gently added into dishes. At specified intervals (30 min and 1, 2, and 4 h), the treatment medium was removed and cells were washed twice with PBS, and fixed with 4% paraformaldehyde (PFA) solution for 10 min at 37°C. Fixed cells were counter-stained with a blue nuclear dye, DAPI, for 30 min at room temperature, and washed with PBS thrice. Coverclips were mounted on microslides, and cells were analyzed with a confocal laser scanning microscope (Zeiss LSM710, Jena, Germany).

### Statistical Analysis

All of the measurement data were displayed as means±standard deviation of the mean (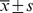). The statistical significance was determined by ANOVA and, when needed, by LSD-*t* test, by using the statistical software SPSS package v13.0. Differences were considered to be significant when *P*<0.05.

## Results and Discussion

### Synthesis and Characterization of Copolymer

**Figure 1:**
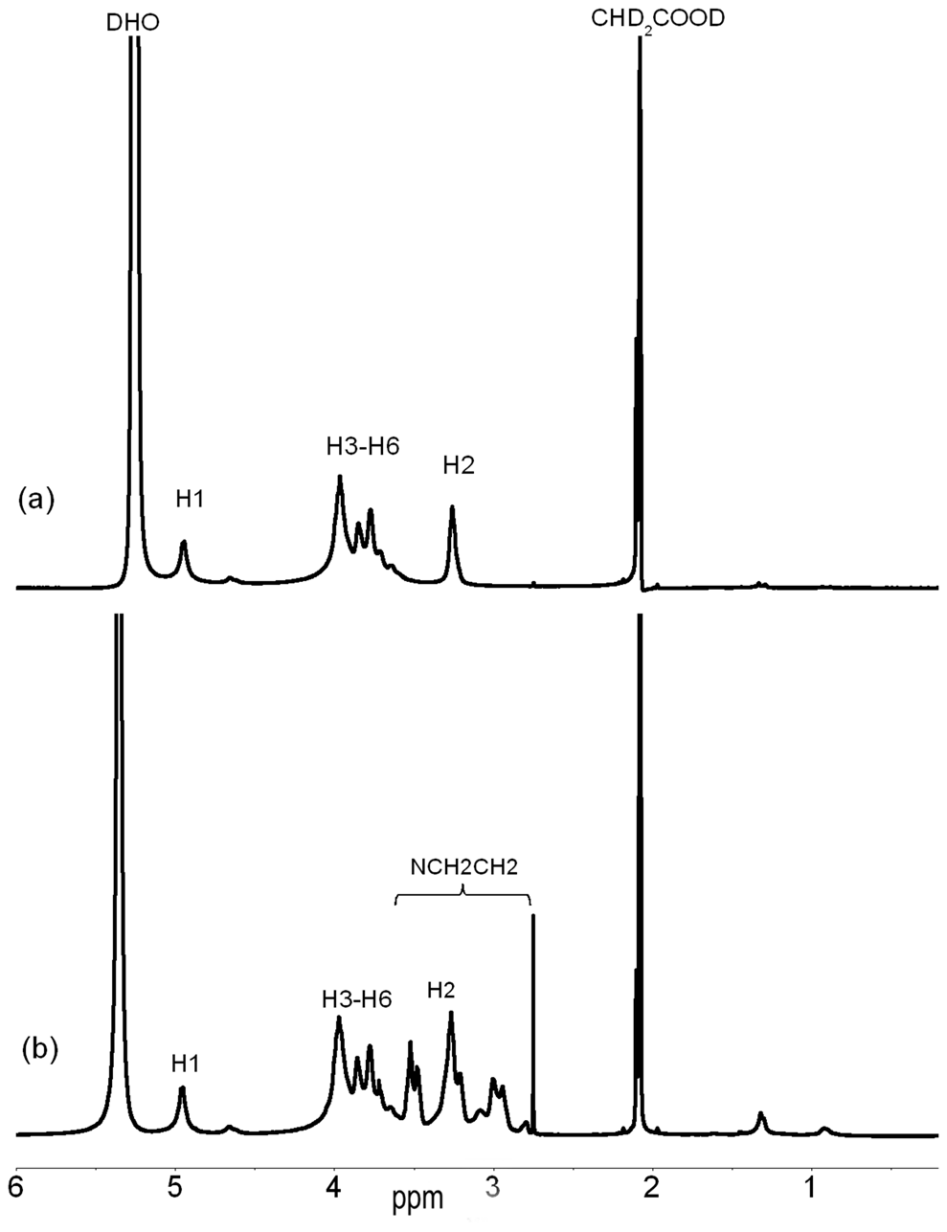
Representative ^1^H NMR spectra of chitosan (CS) and CS-*g*-PEI (CP) in a mixture solution (D_2_O/CD_3_COOD (V_D2O_: V_CD3COOD_ = 1∶1) at 40°C.

**Figure 2:**
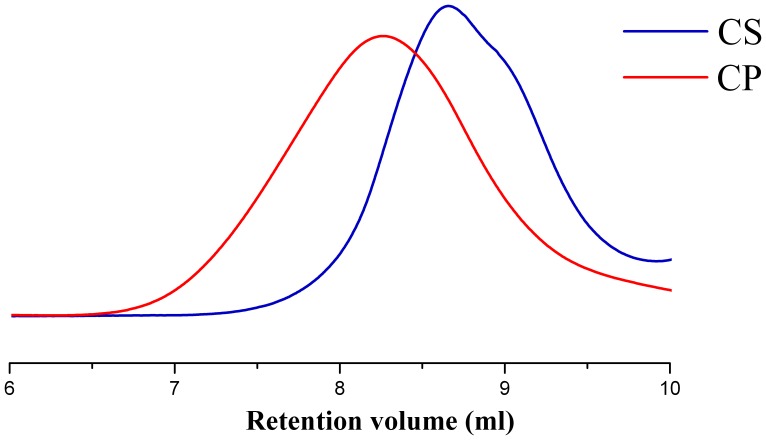
GPC Curves of chitosan (CS) and CS-g-PEI (CP).

The chitosan-graft-polyethylenimine (CP) was synthesized by reaction between CS and PEI with the presence of CDI (Figure S1). During the synthesis of CP, we used excessive PEI, low reactive temperature, and relative slow reaction rate to avoid gelation. Composition of synthesized copolymer was analyzed by ^1^H NMR. As shown in Figure 1, the proton peaks area of CS appeared at 4.94 ppm (H-1 of glucosamine ring), 3.26 ppm (H-2 of glucosamine ring), 3.52–4.31 ppm (H-3, H-4, H-5, H-6 of glucosamine ring). Compared with CS, there were the characteristic chemical shifts of PEI (2.72–3.38 ppm) on the ^1^H NMR spectra of CP, indicating that PEI was grafted to the CS chain [30]. The degree of grafted (DG) of PEI onto CS was calculated by the following formula:
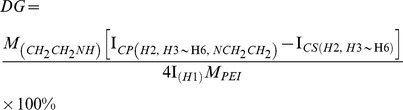
Where ***M***_PEI_ and ***M***_CH2CH2NH_ represents the molecular weight of PEI and -CH_2_CH_2_NH- respectively, I_CP(H2, H3∼H6, NCH2CH2)_ represents the sum of the integral value of correspondent peaks(-CH_2_CH_2_NH-, H-2, H-3, H-4, H-5, H-6 of glucosamine ring on CS chain on CP chain), I_CS (H2, H3∼H6)_ represents the integration values of correspondent peaks (H-2, H-3, H-4, H-5, H-6 of glucosamine ring on CS chain), I_H1_ represents the integration values of H-1, which was defined as 1 during the process of calculation. The DG of PEI was calculated as 7.4% as shown in Table 1. Molecular weights of CS and CS-*g*-PEI (CP) were traced by gel permeation chromatography (GPC). The molecular weight distribution of CP shifted into the shorter retention time, indicating an increase in the molecular weight after grafting PEI onto CS as compared with nascent CS (Figure 2). From the results of GPC (Table 1), the molecular weights (*M*_n_) of CS and CP were 1.98×10^4^ and 4.55×10^4^, respectively, and the DG of PEI onto CS were calculated by the following equation:

**Table 1:** Characteristic of prepared CS-g-PEI (CP).

*M*_n_ of CS	*M*_n_ of CS-*g*-PEI	DG^a^ (mol %)	DG^b^(mol %)
1.98×10^4^	4.55×10^4^	10.9%	7.4%

^a^ calculated from GPC.

^b^ calculated from ^1^H NMR.



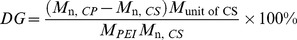

*M*_PEI_ is 1800, and *M*_unit of CS_ = *M*_C6H11O4_×90%+*M*_C8H13O5_×10% = 151.2. The DG of PEI calculated as 10.9% from GPC was different from that calculated as 7.4% from ^1^H NMR, which may result from different measure and calculation methods.

### Physiochemical Characteristics of CP/DNA Nanoparticles

**Figure 3:**
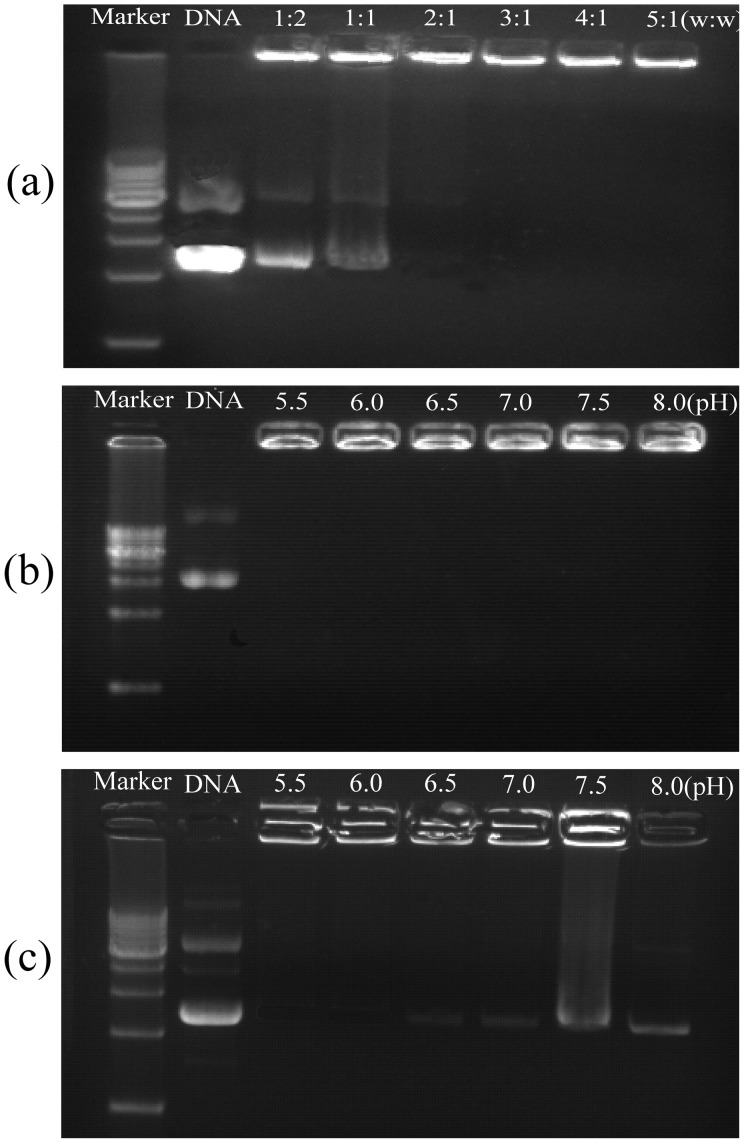
Gel retarding analysis of CP/DNA nanoparticles. Lane 1: DNA marker. Lane 2: naked DNA control. Lane 3–8: CP/DNA nanoparticles prepared at CP:DNA weight ratios of 1∶2, 1∶1, 2∶1, 3∶1, 4∶1, and 5∶1 (a); electrophoresis photo of CP/DNA nanoparticles prepared with CP:DNA weight ratio = 3 at different pH levels (b); electrophoresis of CS/DNA nanoparticles prepared with the CS:DNA weight ratio = 3 at different pH levels (c).

The CP/DNA nanoparticles formation and the CP with DNA condensation capability were evaluated by AGE. Figure 3(a) demonstrates that the DNA migration was completely retarded when CP:DNA weight ratios were around three, indicating DNA bound to CP tightly and completely with little free DNA existing. Figure 3(b) shows that the CP with DNA nanoparticles’ condensation capability at CP:DNA weight ratio = 3 was not affected by pH levels. Figure 3(c) demonstrates that some pDNA escaped from CS/DNA nanoparticles when pH value increased to ≥7.0, indicating that the interaction between pDNA phosphate groups and CS amino groups was dependent on pH level to a certain degree. This is because CS contains primary amine group with a pKa value of about 6.5, at acidic pH<pKa, primary amines in CS backbone become positively charged, and this protonated amine enables CS to bind to negatively charged DNA via an electrostatic interaction. The interaction between the positively charged CS and negatively DNA leads to the nanoparticles formation in the aqueous milieu, preventing pDNA to be released from complexes. However, under either neutral or alkaline condition, where CS is slightly charged, CS cannot completely encapsulate all pDNA [12]. This is quite different from above results with CP as shown in Figure 3(b).

**Figure 4:**
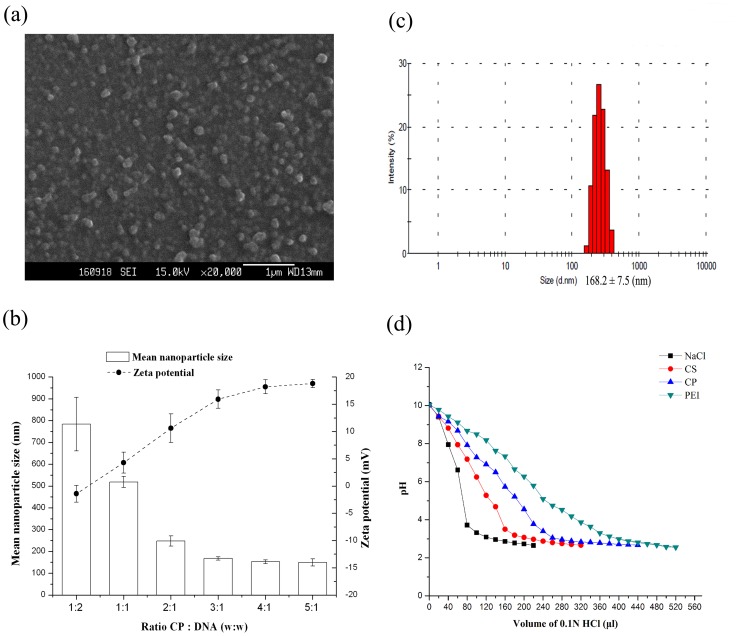
Physiochemical property of CP and CP/DNA nanoparticles. (a) SEM images of CP/DNA nanoparticles at CP:DNA weight ratio = 3; (b) the effect of CP:DNA weight ratios on the particle size and the zeta potential of resulting nanoparticles (n = 3; error bars represent standard deviation); (c) size distribution of CP/DNA complexes prepared at the CP:DNA weight ratio = 3 measured by Mastersizer 2000 laser diffractometer; (d) buffering capacities of PEI (25 kDa), CS, and CP copolymers.

The CP/DNA nanoparticles formation was also monitored by observation of the morphology and their particle size and zeta potential changes as a function of the CP:DNA (w/w) ratio. Figure 4(a) shows a representative SEM image of a CP/DNA complex consisting of spherical particles with diameters of approximately 100–300 nm. Surface properties are important factors that influence the complex uptake by cells. Generally, small particle size and positive surface charge would lead to higher internalization rates [8]. As shown in Figure 4(b), the particle size and zeta potential of nanoparticles were highly dependent on the CP:DNA (w/w) ratio. When the CP:DNA (w/w) ratio was 0.5, the complex size was 583.7±123.0 nm; however, at a ratio of 4, the complex size declined to 152.3±9.1 nm. This could be explained by the fact that the formation of polymer complexes with DNA is through ionic interactions [8], [14], [31]. Additionally, at high CP:DNA (w/w) ratios, can be observed net electrostatic repulsive forces to prevent aggregation among complexes. The relatively homogenous size distributions of nanoparticles, measured by dynamic light scattering, are shown in Figure 4(c). As shown in Figure 4(b), the zeta potential became more positive as the CP amount within the polyelectrolyte complex increased. The zeta potential was negative when CP:DNA (w/w) ratio = 0.5, but with the increasing of the CP:DNA (w/w) ratio the zeta potential rapidly increased to positive values. These results, together with those obtained from gel retardation experiments, suggested that CP/DNA complexes could form positive potential nanoparticles when CP:DNA (w/w) ratio >2. These properties are necessary for CP/DNA nanoparticles to obtain successful gene transfection as the positive surface charge and the small complex size are vital parameters to determine their cellular uptake and interactability with the cell membrane [14], [32].

Figure 4(d) shows the buffering capacity of different polymers in the pH range of 10 to 2.6. It demonstrated that the amount of 0.1 N HCl required in bringing the pH from 10 to 2.6 increased from NaCl, CS, and CP to PEI, showing the maximum buffering capacity of PEI. The endosomal DNA nanoplexes release into the cytoplasm is one of the most important parameters that depend on the intrinsic buffering capacity of the vector. It is interesting to note that CP copolymers exhibits considerable buffering capacity, which would help in release of pDNA from endosomes/lysosomes (proton-sponge effect) [27], [33].

### Cell Viability

**Figure 5:**
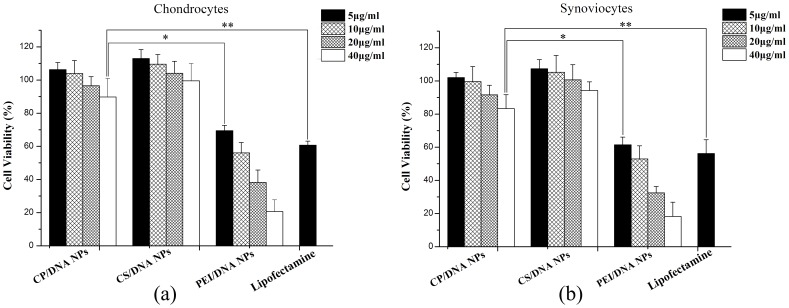
Cell viabilities of CP/DNA nanoparticles, CS/DNA nanoparticles, PEI/DNA nanoparticles, and Lipofectamine™ 2000 in primary chondrocytes (a) and synoviocytes (b). *P<0.01 compared to PEI/DNA nanoparticles; **P<0.01 compared to Lipofectamine™ 2000.

In this study, the cytotoxicity of various nanoparticles (CP/DNA, CS/DNA, and PEI/DNA) was determined by the MTT assay. As the concentration of the CP/DNA nanoparticles increased to 20 µg/ml, it showed a slight increase in cytotoxicity. However, the average cell viability was >90% for both chondrocytes and synoviocytes. Such viability was much higher than that of either PEI (25 kDa)/DNA nanoparticles or Lipofectamine™ 2000, which showed a viability <70% at 5 µg/ml, and even decreased to <40% viability for PEI/DNA nanoparticles at 20 µg/ml (Figure 5). For CP/DNA nanoparticles, when the concentration increased to 40 µg/ml, it showed a slight increase in cytotoxicity, with the cell viabilities being >85% for both chondrocytes and synoviocytes. However, the cell viability was still significantly higher than that of either PEI/DNA nanoparticles (5 µg/ml) or Lipofectamine™ 2000 (5 µg/ml). For cell viability assay on chondrocytes, one-way ANOVA showed statistical significance (*F* = 24.11, *P*<0.01); further analysis with LSD-*t* test revealed that the cell viability of CP/DNA group in 40 µg/ml was significantly higher than that of other two groups (*P*<0.01). Similarly, for cell viability assay on synoviocytes, One-way ANOVA indicated statistical significance (*F* = 19.99, *P*<0.01); further analysis with LSD-*t* test revealed that the cell viability of CP/DNA group in 40 µg/ml were also significantly higher than that of other two groups (*P*<0.01). These results may be related to that low Mw PEI is less toxic, and CS has good biocompatibility and biodegradability [8]–[12]. These cytotoxicity results indicate that CP/DNA nanoparticles should be a safer carrier than PEI/DNA nanoparticles and Lipofectamine™ 2000, and specify a safe range for the CP/DNA nanoparticles (5–20 µg/ml) application to joint tissue/chondrocytes or synoviocytes.

### CP/DNA Complex Transfection Efficiency

**Figure 6:**
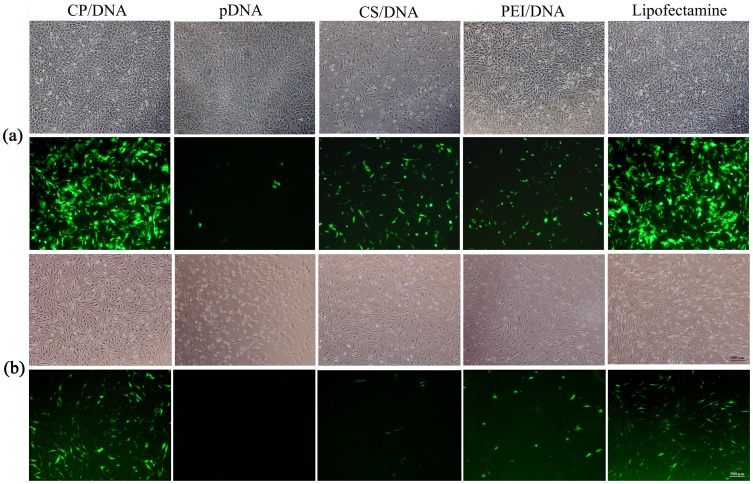
Images of chondrocytes (a) or synoviocytes (b) transfected with CP/DNA nanoparticles, naked pDNA, CS/DNA nanoparticles, PEI (25 kDa)/DNA nanoparticles, and Lipofectamine™ 2000 as observed under fluorescence microscope or inverted phase contrast microscope. (40× magnification for upper panel under inverted phase contrast microscope, and 40× magnification for lower panel under fluorescence microscope).

**Figure 7:**
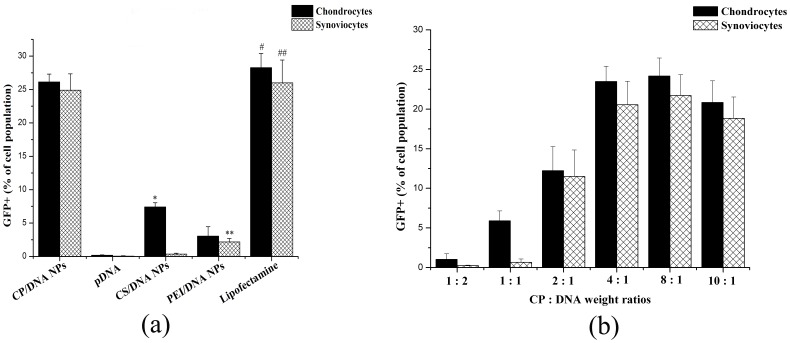
*In vitro* transfection efficiency of CP/DNA nanoparticles. (a) *In vitro* transfection efficiency of CP/DNA nanoparticles in both chondrocytes and synoviocytes compared to that of naked pDNA, CS/DNA nanoparticles, PEI (25 kDa)/DNA nanoparticles, and Lipofectamine™ 2000 (n = 3; 48 h post-transfection; error bars represent standard deviation). *P<0.01 when CP/DNA nanoparticles compared to CS/DNA nanoparticles transfected towards chondrocytes (n = 3); **P<0.01 when CP/DNA nanoparticles compared to PEI (25 kDa)/DNA nanoparticles transfected towards synoviocytes (n = 3); # or ## *P*>0.05 when CP/DNA nanoparticles compared to Lipofectamine™ 2000 transfected towards chondrocytes or synoviocytes (n = 3). (b) Percentage of chondrocytes or synoviocytes transfected *in vitro* using CP/DNA nanoparticles as measured by flow cytometry 48 h post-transfection. The influence of CP:DNA weight ratios on the transfection efficiency was assessed 48 h post-transfection (n = 3; error bars represent standard deviation).

In the present study, we compared the transfection efficiency of CP/DNA nanoparticles with that of CS/DNA nanoparticles, naked pDNA, PEI (25 kDa)/DNA nanoparticles, and Lipofectamine™ 2000. Figures 6 and 7(a) show that the transfection efficiency of CP/DNA complex was similar to that of the Lipofectamine™ 2000 at CP:DNA (w/w) ratio = 3, and significantly higher than that of CS/DNA nanoparticles, PEI (25 kDa)/DNA nanoparticles, and naked pDNA [Figs. 7(a) and S2], which is consistent with some previous studies [14], [34]. The transfection efficiency of different groups were analyzed by one-way ANOVA, which demonstrated statistical significance for chondrocytes (*F* = 21.88, *P*<0.01); further analysis with LSD-*t* test revealed that the transfection efficiency of CP/DNA nanoparticles was significantly higher than that of the naked plasmid, PEI (25 kDa)/DNA, and CS/DNA nanoparticles (*P*<0.01). However, there were no significant differences between CP/DNA nanoparticles and Lipofectamine™ 2000 (*P*>0.05). Similarly, for synoviocytes, one-way ANOVA demonstrated statistical significance (*F* = 152.825, *P*<0.01); further analysis with LSD-*t* test revealed that the transfection efficiency of CP/DNA nanoparticles was higher than that of the naked plasmid, PEI (25 kDa)/DNA and CS/DNA nanoparticles (*P*<0.01). However, there were no significant differences between CP/DNA nanoparticles and Lipofectamine™ 2000 (*P*>0.05). Jiang *et al*. [14] reported that the transfection efficiency of CP/DNA copolymer is cell-type dependent. However, in the present study no significant differences were observed between chondrocytes and synoviocytes, situation that was also quite different from either CS/DNA nanoparticles or HA-modified CS/DNA nanoparticles in our previous study [16]. It is known that a key cellular barrier impeding the transfection efficiency of non-viral gene vectors is the inefficiency release of endosomally trapped DNA into the cell cytosol [4], [5], [8], [9]. PEI is known to possess a very good buffering capacity [21], which has also been confirmed in the present study, and could escape from endosome through the proton-sponge mechanism, thus facilitating gene entry into the nucleus [8], [9]. However, PEI also exhibits Mw-dependent transfection efficiency: PEIs with high Mw have better gene transfer capability, but suffer from charge associated toxicity. PEIs with low Mw are non-toxic, but have poor transfection efficiency [9], [20], [34]. In the present study, we deliberately choose low Mw of PEI graft with CS to form CP complexes. The total amount of amine content in complex is close to PEI (25 kDa), which allows complexes easily to escape from endosome due to a higher buffering capacity, and simultaneously to achieve high transfection efficiency due to its low cytotoxicity.

Another prerequisite to achieve successful transfection is that the bound pDNA must release from complexes: the unpacking of DNA from the polymeric vector is an important and crucial step in the transfection mediated by polymers [35]. CS has been reported to interact with pDNA via either strong electrostatic/hydrogen bonding or hydrophobic interactions [36]. High Mw PEIs also show high binding capacity with pDNA [37]. The binding ability has been found to be in the order CS>>>PEI (25 kDa)>CP>PEI (2.5 kDa) [9]. These crucial observations suggest that the CP copolymer binding capability is neither strong nor weak, which may be beneficial for improving transfection efficiency. CP/DNA nanoparticles’ transfections showed a delayed EGFP expression, which was quite different from that of Lipofectamine™ 2000. At 24 h post-transfection, both CP/DNA and CS/DNA nanoparticles expressed weak EGFP. For its part, in cells transfected with naked pDNA, almost no expression of EGFP was observed. At 48 h, the EGFP-expression increased significantly in the CP/DNA nanoparticles group, and its fluorescence intensity also increased significantly compared to that of CS/DNA nanoparticles, PEI (25 kDa)/DNA nanoparticles, and naked pDNA [Figs. 6 and 7(a)]. The CS sustained-release property leads to relatively retardant release of pDNA from nanoparticles, which may explain why the expression of foreign transfected genes remained lower initially, the transfection efficiency increased after prolonged time, and the expression lasted much longer, which was different from that with Lipofectamine ™ 2000, associated with a rapid expression [16], [38].

Figure 7(b) shows that the transfection efficiency of CP/DNA complex increased with the increasing of CP:DNA (w/w) ratio, and appeared a plateau after CP:DNA (w/w) ratio = 4. The transfection efficiencies of CP/DNA nanoparticles for chondrocytes of different CP:DNA (w/w) ratios were analyzed by one-way ANOVA, which demonstrated statistically significant difference (*F* = 62.07, *P*<0.01); further analysis with LSD-*t* test revealed that, when the CP:DNA weight ratio reached 8, the transfection efficiency of CP/DNA nanoparticles was significantly higher than that when CP:DNA weight ratio was 0.5 to 2 (*P*<0.01). Similar results were obtained for synoviocytes. With the increasing CP amount, the content of grafted PEI (1.8 kDa) helped complex to escape from endosome more easily, and it will not cause cytotoxicity to either chondrocytes or synoviocytes. On the other hand, as the CP:DNA (w/w) ratio increased, the copolymer size became smaller, and surface charge became more positive, thus leading to higher complexes internalization rates. This also would facilitate the higher transfection efficiency. However, when CP: DNA (w/w) ratio was greater than 8, it implies an considerable increase in the CP concentration in the complex, with too higher positive charge of the polyplexes yielding an overly stable complexes with anion DNA which leads to hard dissociation between CP and DNA, and as a consequence, the efficiency of plasmid release may decrease, thus showing reduced transfection [12], . In addition, with the CP concentration became too high, as shown in Figure 5, its cytotoxicity was also increased slightly, which would affect the transfection efficiency and even conversely lead to decreased gene transfection [14], [41]. As a result, the transfection efficiency of CP/DNA complex appeared a plateau after CP:DNA (w/w) ratio = 4.

### CP/DNA Complex Intracellular Trafficking

**Figure 8:**
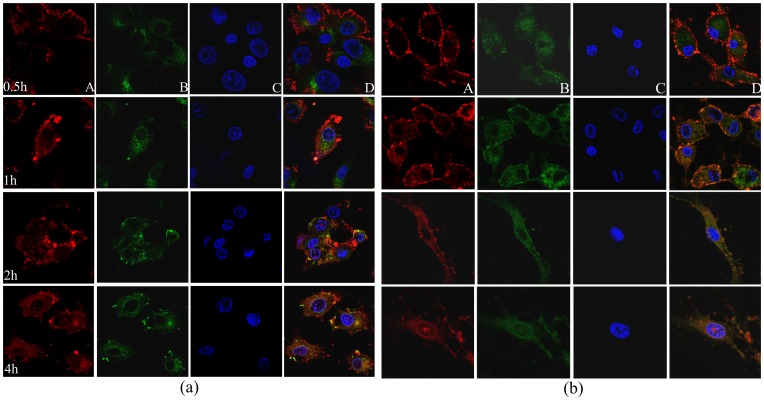
Intracellular distribution of Cy3-labeled pDNA/CP complexes was observed with a confocal fluorescence microscope in chondrocytes (a) and synoviocytes (b). (Panel 1) 0.5 h post-incubation; (Panel 2) 1 h post-incubation; (Panel 3) 2 h post-incubation; and (Panel 4) 4 h post-incubation. Row A shows the Cy3-labeled pDNA (red); row B shows the lysosomal (green); row C shows the nucleus (blue); and row D shows the overlap of A, B, and C rows content.

To investigate the cellular entry path, Cy3-labeled pDNA (red) CP/DNA was simultaneously added to both chondrocytes and synoviocytes, LysoTracker Green DND-26 was added to label lysosomes, and cells were fixed with PFA at specified intervals. We observed that CP was able to carry pDNA inside cells (cytoplasm) within 30 min to 1 h after the addition of nanoparticles to the cells. Further, pDNA could be observed inside the nucleus 2 h post addition of complexes, and a greater pDNA mount could be detected in nucleus after 4 h (Fig. 8). These observations clearly indicated that CP copolymers are capable of efficiently carrying the desired gene inside both chondrocytes and synoviocytes. In contrast, as Feng *et al*. [42] reported previously, PEI (25 kDa)/DNA nanoparticles could escape from endosome quickly, and the pDNA carried by nanoparticles detached from these and quickly localized in HeLa cells nuclei. However, the pDNA carried by CS was difficult to enter the nucleus even after 6 h of the CS/DNA complexes incubation [43], suggesting that it might take more time for CS-based polyplexes to escape from endosomes, and to undergo uncoupling of CS/DNA complexes than PEI polyplexes [12]. It has been suggested that, if polyplexes escape from the endosome too fast, it may lead to a large number of endosome ruptures in a short term, thus causing damage to the cell physiological environment and increasing the cytotoxicity [8]. In the present study, we have shown that the CP/DNA complex could escape from endosomes at a proper rate, simultaneously resulting in increased transfection efficiency and reduced cytotoxicity.

## Conclusions

In the present study, CP/DNA nanoparticles were created as a novel, non-viral gene carrier targeted to OA and other joint diseases. The particle size and zeta potential of CP/DNA nanoparticles were related to the CP:DNA (w/w) ratio: there was a decrease in size and an increase in the surface charge with increasing of CP:DNA (w/w) ratio. The CP buffering capacity was found to be significantly enhanced compared to that of the CS. The transfection efficiency of CP/DNA nanoparticles was significantly higher than that of CS/DNA nanoparticles, PEI (25 kDa)/DNA nanoparticles and naked pDNA, and was similar to that of the Lipofectamine™ 2000 towards articular chondrocytes and synoviocytes. The *in vitro* transfection efficiency of CP/DNA nanoparticles was found to be dependent on the CP:DNA (w/w) ratio. The average cell viability post-treatment with CP/DNA nanoparticles was >90% for both chondrocytes and synoviocytes, even when nanoparticles dose was increased to 20 µg/ml. This viability was much higher than that of PEI (25 kDa)/DNA nanoparticles. Intracellular trafficking studies found that CP copolymers were capable of efficiently carrying the pDNA inside both chondrocytes and synoviocytes, and the pDNA could be detected entering into the nucleus post 4 h incubation.

These results suggest that CP/DNA nanoparticles might be a safe and efficient non-viral vector for gene delivery to both chondrocytes and synoviocytes. Further studies should focus on evaluating the *in vivo* application of these novel CP/DNA nanoparticles in the treatment for joint diseases such as OA.

## Supporting Information

Figure S1 **Schematic representation of preparation of CP copolymers.**
(TIF)

Figure S2 **EGFP fluorescent intensity of cells from different groups. (n = 3; 48 h post-transfection).**
(TIF)
