# Supplementary figures and images for: Chitosan-Graft-Polyethylenimine/DNA Nanoparticles as Novel Non-Viral Gene Delivery Vectors Targeting Osteoarthritis
Source: PLoS One. 2014 Jan 2;9(1):e84703. doi: 10.1371/journal.pone.0084703 (PMC3879331; doi:10.1371/journal.pone.0084703)

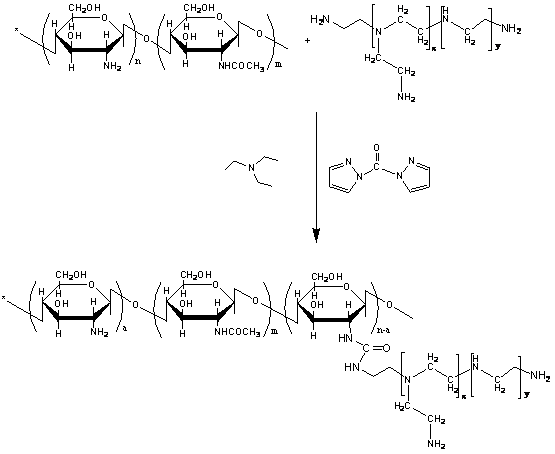

Supplement: Figure S1 — Schematic representation of preparation of CP copolymers. (TIF) [file pone.0084703.s001.tif]

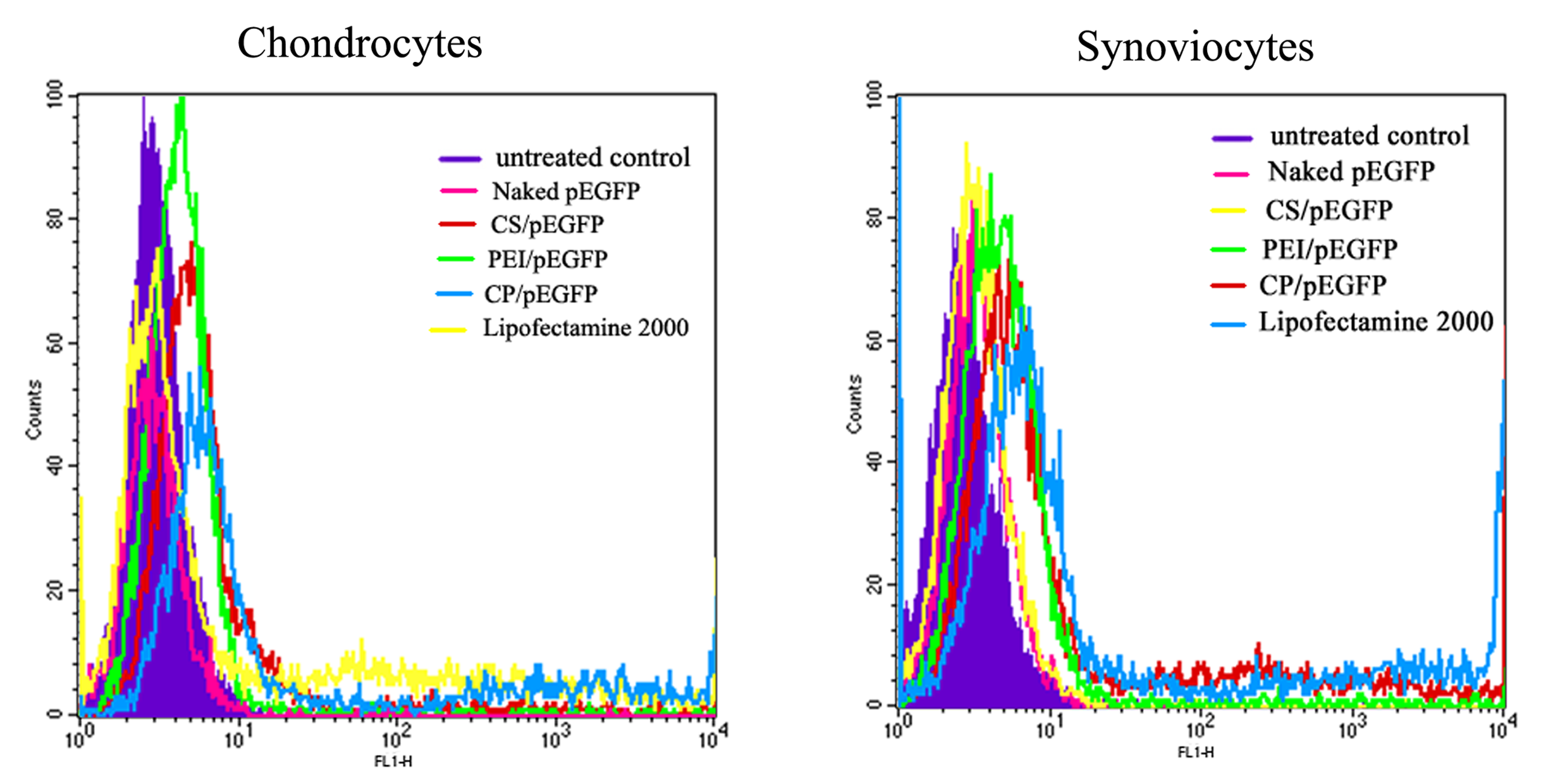

Supplement: Figure S2 — EGFP fluorescent intensity of cells from different groups. (n = 3; 48 h post-transfection). (TIF) [file pone.0084703.s002.tif]
